# Trends in prevalence of diarrhoea, Kaposi’s sarcoma, bacterial pneumonia, malaria and geohelminths among HIV positive individuals in Uganda

**DOI:** 10.1186/s12981-015-0060-0

**Published:** 2015-06-13

**Authors:** John Rubaihayo, Nazarius M Tumwesigye, Joseph Konde-Lule

**Affiliations:** Department of Epidemiology and Biostatistics, School of Public Health, College of Health Sciences, Makerere University, Kampala, Uganda; Department of Public Health, School of Health Sciences, Mountains of the Moon University, Fort Portal, Uganda

## Abstract

**Background:**

Trends in prevalence of opportunistic infections (OIs) associated with the human immunodeficiency virus (HIV) in resource poor settings have previously not been well documented. The objective of this study was to describe the trends in prevalence of Diarrhoea, Bacterial pneumonia, Kaposi’s sarcoma, Malaria and Geohelminths among HIV positive individuals over a 12 year period in Uganda.

**Methods:**

Observation data for 5972 HIV positive individuals enrolled with the AIDS support organisation (TASO) in Uganda were analysed. Study participants were drawn from three HIV clinics located in different geographical areas of Uganda and followed from January 2002 to December 2013. The prevalence trends for the above OIs were plotted using the Box Jenkins moving average technique. *X*^2^-test for trend was used to test for the significance of the trends and Pearson’s correlation coefficient used to test for the strength of linear relationship between OI prevalence and calendar time. Mixed effect linear regression was used to estimate average monthly change in prevalence with monthly variation modelled as a random effect.

**Results:**

A total of 204,871 monthly medical reports were retrieved and analysed. 73 % (4301/5972) were female with a median age of 32 years (inter-quartile range 26–39). Overall, significant decreasing mean annual prevalence trends (p < 0.05, X^2^_trend_) were observed for Diarrhoea (<1 month) with Pearson’s correlation coefficient (r = −0.89), Malaria (r = −0.75), Bacterial Pneumonia (r = −0.52), and Geohelminth (r = −0.32). Non-significant increasing mean annual prevalence trend was observed for Kaposis sarcoma (p = 0.20, X^2^_trend_; r = +0.26). After adjusting for age, sex and clinic in a mixed effects linear regression model, average monthly prevalence declined significantly at a rate of 0.4 % for Kaposis sarcoma, 0.3 % for Geohelminths, 2 % for Malaria, 1 % for Bacterial Pneumonia and 3 % for Diarrhoea(<1 month). However, the rate of decline per month differed significantly (p < 0.05) by HIV clinic for Diarrhoea (<1 month), and age, sex and clinic for malaria.

**Conclusions and recommendations:**

Overall, decreasing trends were observed in the above OIs. However the trends differed significantly by OI, geographical location and demographic characteristics. There is urgent need to integrate interventions targeting malaria and geohelminths in HIV programmes.

## Background

Several African countries with a generalised HIV epidemic are struggling with availing lifelong ARVS to an increasing population of eligible HIV patients amidst scarce resources. According to the World health Organization [1], 9.7 million persons living with HIV in low and middle income countries had access to highly active antiretroviral therapy by the end of 2012, and AIDS-related deaths were reduced to about 1.6 million compared to 2 million deaths attributed to AIDS in 2002 [2]. Uganda is one of the few sub-Saharan countries in which the magnitude of the HIV epidemic has been substantially reduced and stabilized in the past decades; though recent reports show a slight increase in HIV prevalence among adults from a national average of 6.4 % in 2005 to 7.3 % by 2011 [3]. According to the Uganda AIDS indicator survey (2011), the burden of HIV differs by geographical area with central Uganda having the highest prevalence of HIV (10.6 %), followed by mid-northern (8.3 %) and then mid and south western (8–8.2 %) and lowest prevalence in mid-eastern(4.1 %) [3] but was not clear whether the burden of HIV related morbidity and mortality follows the same pattern.

Access to highly active antiretroviral therapy (HAART) in Uganda has been steadily increasing since HAART was introduced in 2004 and by the end of 2013, 69.4 % (570,373/821,721) of eligible adults and children had access to HAART; though this was still below the 80 % national target by 2015 [4]. Co-trimoxaxole (TMP-SMX) prophylaxis has also been shown to prevent or significantly reduce the incidence of opportunistic infections and related mortality in HIV-infected individuals [5–8] . In Sub-Saharan Africa where access to HAART is still limited, cotrimoxazole prophylaxis remains the most viable alternative and has greatly increased the chances of survival for HIV-infected individuals who are eligible but cannot access HAART [9]. UNAIDS recommends cotrimoxazole prophylaxis for life to all persons living with HIV/AIDS regardless of their immunological status and whether they are on HAART or not [10]. However, even with increasing availability of HAART and co-trimoxazole (TMP-SMX) prophylaxis, life threatening opportunistic infections remain the most important aspect of care for persons living with HIV associated with stigma, poor quality of life and high medical care costs. Opportunistic infections therefore have greatly contributed to poverty among those infected and affected by HIV hence an impediment to the attainment of the millennium development goals (MDGs) on health and poverty eradication particularly in resource poor countries [4]

In Uganda, universal cotrimoxazole prophylaxis was introduced in 2003 in all public and private health facilities providing care and treatment to persons infected with HIV/AIDS [11]. The AIDS support Organisation (TASO), one of the oldest and largest non-governmental organisation (NGO) providing treatment and care to persons living with HIV/AIDS in Uganda, rolled out universal co-trimoxazole prophylaxis in early 2003 and HAART in 2004. However, it was not known whether TASO HIV/AIDS care and treatment programme had any significant effect on trends of opportunistic infections or not. Trends of OIs in Uganda have previously not been fully examined due to lack of sufficient data. Trends of OIs provide invaluable information that can be used to evaluate the impact of treatment and preventive interventions over time and can also help to inform planning and policy on future preventive and treatment strategies in resource poor settings. We analysed trends in prevalence of diarrhoea, bacterial pneumonia, kaposi sarcoma, malaria and geohelminths among HIV positive individuals enrolled with TASO HIV/AIDS care and treatment programme from 2002 to 2013 inclusive. The trends of others OIs (TB, cryptococcosis, candidiasis, genital ulcer and herpes zoster) were also analysed but were considered elsewhere. These OIs were selected on the basis that they are the commonest OIs in Uganda and are easy to diagnose.

## Methods

### Study design

Observational data for 5972 HIV positive individuals enrolled with the AIDS support organisation (TASO) in Uganda were retrieved and analysed. The study period was from January 2002 to December 2013 and the opportunistic infections of interest were diarrhoea, bacterial pneumonia, kaposi sarcoma, malaria and geohelminths. Kaposis sarcoma though strictly speaking is not an opportunistic infection but was included because of being associated with opportunistic viral infections ((human herpes virus type 8) [12]. The 12 year review period was categorised into three time periods that signify important milestones in the prevention and treatment of opportunistic infections associated with HIV/AIDS in Uganda. First period covered 2 years immediately prior to HAART rollout in Uganda (2002–2003). Second period is from 2004 to 2008 denoted as “Early HAART” in which there was very limited access to HAART only available to a few severely ill HIV positive individuals (CD4 cell count <200cells/μl of blood). The third period is from 2009 to 2013 denoted as “Late HAART” in which HAART access was extended to include even HIV+ individuals with CD4 cell equal to 350cells/ μl to of blood. Data were analyzed using autoregressive moving average time series and multilevel mixed effects linear regression models adjusting for potential confounders whose data was available.

### Study settings

Data for the study were obtained from TASO electronic data bases. TASO is one of the oldest and largest HIV/AIDS care programme in Uganda and sub-Saharan Africa [13]. The organisation which was started in 1987 has 11 HIV clinics spread across Uganda and has accumulated data on HIV/AIDS for the last 3 decades and thus provided a good opportunity to examine trends of common and rare opportunistic infections associated with HIV/AIDS in Uganda. TASO HAART programme started as part of the National HAART roll-out programme in public health facilities in 2004. Being one of the largest HAART providers in the country, TASO attracted a lot of support from different funders supporting HAART programmes in sub-Saharan Africa including the President’s Emergency Plan for AIDS Relief (PEPFAR) and the Global Fund to Fight AIDS, Tuberculosis and Malaria. Initially, HAART eligibility was based on WHO 2006 guidelines i.e. WHO stage 3 or 4 illness or a CD4 cell count < 200 cells/μl for adults and adolescents and WHO stage III, advanced stage II or stage I with CD4 cell percentage less than 20 % for children more than 18 months of age [14]. However, in 2010 new HAART guidelines [15] that raised the threshold for adults and adolescents to a CD4 cell count >200 cells/μl but ≤350 cells/μl or WHO clinical stage3 or 4 irrespective of CD4 cell count were adopted [16]. Those who were not eligible for HAART were offered cotrimoxazole prophylaxis. All HIV positive patients were usually screened for tuberculosis (TB) at admission and at HAART initiation. HIV-1 viral load monitoring was not used in theses settings. Given the resource constraints and pressing clinical needs, HIV positive patients could be initiated on HAART basing on clinical presentation without CD4 evaluations. The TASO HAART programme has a robust community counselling and social support component including provider initiated and voluntary HIV testing and counselling, monitoring adherence to ART, assessing general clinical conditions of their clients and reporting those severely ill or dead. All services are free of charge including anti-retroviral drugs (ARVs) for those who are eligible [17].

### Sampling and sample size

Three TASO HIV clinics were purposively selected basing on volume, quality of data and geographical representation. The HIV clinics selected were TASO Mulago HIV clinic in central Uganda, TASO Mbarara HIV clinic in south-western Uganda and Tororo HIV clinic in Eastern Uganda. All HIV positive adults (15 years and above) who were in care at the three selected HIV clinics from 1st January 2002 were included in the study.

### Data collection

The data were collected by TASO medical staff following an established protocol for all TASO HIV clinics. In brief, each client was expected to attend the clinic at least once a month. At each clinic visit, data per client was collected on a standardized medical form detailing the client’s demographic information, clinical condition, medical history, OI diagnosis, ART use and level of adherence, prophylaxis use, any other treatment given and side effects/toxicities if any. Diagnosis of the different disease conditions was based on the Ugandan Ministry of Health and WHO case definition guidelines [18]. Bacterial pneumonia was clinically defined as acute onset of fever, cough, dyspnoea associated with chest pain and responsive to antibiotics; diarrhoea < 1 month was clinically defined as three or more loose or liquid bowel movements per day lasting for a period not exceeding 1 month; malaria was clinically defined as fever, body temperature >37 °C and with a laboratory confirmed positive malaria blood slide or a positive rapid diagnosis test (RDT); Kaposi’s sarcoma was clinically defined as persistent skin lesions developing into plaques or nodules; geohelminthiasis was defined as laboratory confirmed positive stool slide for the eggs of any of the following: *Ascaris lumbricodes*, *Trichuris trichiura*, *Necator americanus*, *Strongyloides stercoralis* or *Ancylostoma duodenale.*

Both clinical and laboratory Data were then compiled and entered into the TASO electronic data base by TASO data administrator using EPIINFO vs3 in Access format. Monthly medical data for each participant covering the period January 2002 to December 2013 were extracted by the data administrator, delinked from overt identifiers and then handed over for analysis.

### Data management and analysis

Data cleaning, validation and analysis were done using STATA 12(Stata Corp, Collage station, Texas). Baseline demographic data was summarised and presented by frequency distribution. To analyse trends, data on each OI was assembled and summarised by year and month. Monthly prevalence was calculated from the total number of clients recorded with an event in a month divided by the total number that attended the clinic in that particular month. Annual prevalence per OI was calculated from the mean of the monthly prevalences in a year. A time plot was generated using the Box Jenkins moving average smoothening technique [19]. The moving average smoothing technique achieves this by replacing each element of the time series by *n* neighbouring elements, where n is the width of the smoothening window. We used a centred moving average including 3 observations before and 2 observations after the current observation inclusive, and then computed the mean value as the centred moving average. Pearson’s correlation coefficient was used to test for the strength of linear relationship between OI prevalence and calendar time.

To test for the significance of annualised trends, a *X*^2^-test for trend [20, 21] was used. The trend was considered significant if *X*^2^-trend < 0.05 [21]. To test for the significance of the difference between trends, we used kruskal wallis test [22]. To estimate average monthly change and the effect of covariates, we used mixed effect linear regression analysis with monthly variation modelled as a random effect adjusting for all available potential confounders.

### Ethical considerations

The study obtained ethical clearance from Makerere University School of Public Health Higher Degrees Research and Ethics committee and the Uganda National Council for Science and Technology. Informed consent from study participants was not required as this was routinely collected operational data and the above ethical committees waived the need for consent. However, written consent was obtained from TASO for conducting study and publication of findings with any accompanying images.

## Results

### Baseline characteristics

A total of 204,871 monthly medical reports were analysed. 73 % (4301) were female with a median age of 32 years (inter-quartile range 26–39). More than half of the study participants were subsistence farmers (56 %) with majority having only primary level education (50 %) and 30 % were widowed (Table 1). By 2004, only 9 % of these had access to HAART compared to more than 90 % on HAART in 2013. The overall median age at HAART initiation was 44 years (inter-quartile range 37–50) but was significantly lower for women at 43 (inter-quartile range 37–49) than that of men at 45 (inter-quartile range 40–52) (p = 0.001) (Table 3). Baseline WHO clinical stage, CD4 count and HAART regimen were not significantly different by gender (p > 0.05) (Table 3). The median CD4 cell count at the start of HAART was 128 cells (inter-quartile range 55–190) and the commonly used HAART regimes were zidovudine plus lamivudine and nevirapine (ZDV + 3TC+ NVP) (44 %), followed by stavudine, lamivudine and nevirapine (d4T + 3TC + NVP) (40 %), and other regimen combinations that includes efavirenz (EFV), Tenofovir (TDF) and Lopinavir/ritonavir, etc. were rarely used in this cohort (20 %) (Table 2). Only 3 % of the study participants were on cotrimoxazole/dapson prophylaxis in 2002 compared to 98 % in 2013

**Table 1:** Baseline characteristics of the cohort at the start of the study period (2002), total and clinic-specific

Variable	Total cohort	Tororo	Mulago	Mbarara
	*n(%)*	HIV clinic	HIV clinic	HIV Clinc
		*n(%)*	*n(%)*	*n(%)*
**Sex (n = 5,972)**				
**Female**	4,301(73)	1,071 (71)	1,368 (76)	1,862 (70)
**Male**	1,671 (27)	433 (29)	436 (24)	802 (30)
**Median age (IQR) (n = 5964)**	32 (26,39)	33 (28,40)	30(25,36)	32 (27,39)
**Occupation (n = 5031)**				
**paid employee**	627(12)	120(8)	232(18)	275(12)
**self employed**	1125(22)	271(18)	336(27)	518(23)
**subsistence farmer**	2808(56)	932(62)	563(45)	1313(58)
**others**	471(9)	196(13)	127(10)	148(7)
**Education (n = 5,005)**				
**None**	1199(23)	414(27)	135(11)	650(29)
**Primary**	2521(50)	789(52)	623(50)	1106(49)
**Secondary**	1063(21)	286(19)	415(33)	364(16)
**Tertiary or above**	220(4)	22(1)	82(6.5)	116(5)
**Marital status (n = 5029)**				
**Single (never married)**	195(4)	24(2)	98(8)	73(3)
**Married**	2074(21)	690(45)	441(35)	943(42)
**Divorced**	678(13)	152(10)	324(26)	202(9)
**Widowed**	1504(30)	462(30)	236(19)	806(36)
**Others**	578(11)	191(13)	159(13)	228(10)

**Table 2:** Mean number of study participants who were in care and those accessing HAART each year

Year	Total number in care	Number LFU/died(−) or returning for care(+) n(%)	Total on ART n(%)	Tororo HIV clinic n(%)		Mulago HIV clinic n(%)		Mbarara HIV clinic n(%)	
				In care	On ART	In care	On ART	In care	On ART
**2002**	5972	-	**-**	**-**	**-**	**-**	**-**	-	**-**
**2003**	4725	1247(−21)	**-**	**-**	**-**	**-**	**-**	-	**-**
**2004**	3896	829(−18)	371(9)	1181	-	1185	252(21)	1530	119(8)
**2005**	3551	345(−9)	837(23)	1261	61(5)	1157	343(30)	1133	433(38)
**2006**	2980	571(−16)	934(32)	972	71(7)	931	392(42)	1077	471(44)
**2007**	2401	579(−19)	1051(43)	863	84(10)	686	438(64)	852	529(62)
**2008**	2087	314(−13)	1135(54)	823	101(12)	581	466(80)	683	568(83)
**2009**	2127	42(+2)	1281(60)	887	176(20)	579	497(86)	661	608(92)
**2010**	2190	63(+3)	1693(77)	888	557(63)	570	514(90)	632	622(98)
**2011**	2002	188(−9)	1741(87)	689	577(84)	558	524(94)	655	640(98)
**2012**	1724	278(−14)	1724(100)	578	578(100)	515	515(100)	631	631(100)
**2013**	1772	48(+3)	1772(100)	592	592(100)	533	533(100)	647	647(100)

LFU = Lost to follow up

### Prevalence and trends of OIs

**Fig. 1:**
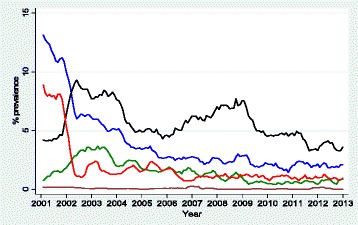
Plot of monthly prevalence for bacterial pneumonia (green line), malaria (red line), diarrhea <1 month)(blue line), geohelminths(black line) and kaposi’s sarcoma (maroon line) over a period of 12 years (2002–2013) expressed as a proportion of HIV-positive persons diagnosed with a particular OI out of the total number who turned up for care per month for the period January 2002 to December 2013

**Fig. 2:**
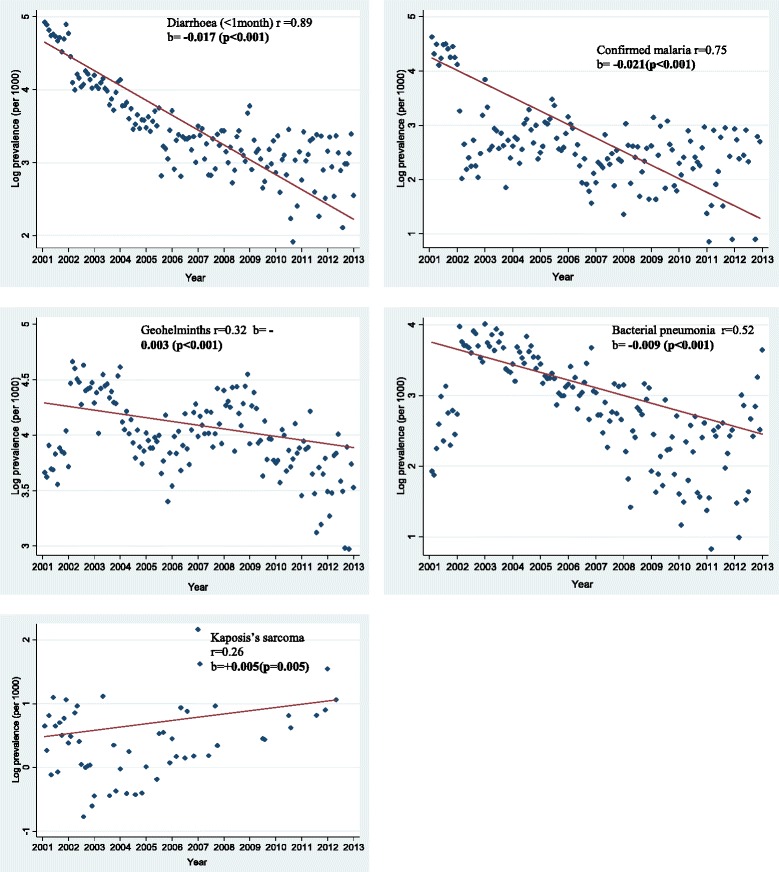
Scatter plot and fitted regression line for monthly prevalence over time (in months) for each OI expressed as a proportion of HIV-positive persons diagnosed with a particular OI out of the total number who turned up for care per month for the period January 2002 to December 2013

Overall monthly trends for each OI are shown in Fig. 1. Best-fit regression lines, R^2^ and p-values for each OI are shown in Fig. 2. Demographic characteristics are shown in Table 1 while study participants accessing HAART over time are shown in Table 2. Background characteristics at the commencement of HAART are shown in Table 3. Average annual prevalence by each OI and statistical tests for trend are shown in Table 4. Average monthly changes in OI prevalence over time after controlling for age, sex and clinic as fixed effects and monthly clustering as a random effect are shown in Table 5.

**Table 3:** Baseline characteristics of the study participants at commencement of HAART, total and gender segregated

Characteristic	Total	Female	Male	p-value*
	(N = 1741)	(N = 1413)	(N = 328)	
**Age in years, median (IQR)**	44(37,50)	43(37,49)	45(40,52)	0.001
**WHO stage III & IV, n (%)**	1113(63.9)	929(65.7)	184(56.1)	0.121
**CD4 cells/μ** **l, median (IQR) (2005–6)**	128(55,190)	144(65,191)	49(49,92)	0.074
**ART regime (2005–2013), n (%)**				
**d4T + 3TC+ NVP**	693(40)	638(45)	136(41)	0.233
**ZDV +3TC + NPV**	774(44)	562(40)	131(40)	
**Other** ^**^	274(16)	213(15)	61(19)	

*Chi^2^-Test

**includes Tenofovir(TDF), Lopinavir/ritonavir, etc.

**Table 4:** Test for trend of mean annual prevalence (per 1000) with calendar time (2002–2013)

	Before HAART		Early HAART					Late HAART					
OI	2002	2003	2004	2005	2006	2007	2008	2009	2010	2011	2012	2013	*X* ^*2*^_*trend*_ *(p-value)*
**Diarrhoea (<1 month)**	117.6	64.2	54.6	37.7	31.6	25.8	25.5	25.9	20.9	18.4	22.0	20.2	177.6 (<0.001)
**% change**	-	−45	−15	−31	−16	−18	−1	+1	−19	−12	+19	−1	
**Kaposis sarcoma**	2.1	1.5	1.5	1.0	1.5	4.5	3.5	-	1.5	2.1	3.1	2.7	1.67 (0.197)
**% change**	-	−29	0	−33	+50	+200	−22	-	-	+40	+48	−13	
**Bacterial Pneumonia**	13.2	32.3	30.1	22.4	15.9	16.7	13.3	9.8	5.8	5.4	5.8	7.7	21.7 (<0.001)
**% change**	-	+145	−7	−26	−29	+5	−20	−26	−41	−7	+7	+33	
**Malaria**	78.7	16.0	15.2	16.7	19.6	10.5	10.2	11.3	11.4	11.0	9.4	9.6	102.6 (<0.001)
**% change**	-	−80	−5	+10	+17	−46	−3	+11	+1	−3	−14	+1	
**Geohelminths**	43.9	87.9	81.8	54.3	47.3	54.4	62.5	71.6	54.9	45.8	40.9	36.6	6.2 (0.0124)
**% change**	-	+100	−7	−34	−13	+15	+15	+15	−23	−16	−11	−10

**Table 5:** Mixed effects linear regression of monthly prevalence (per 1000) and calendar time (months) adjusted for fixed effects (age, sex and clinic) and random effects (monthly clustering)

	Diarrhoea < 1 month^1^ β (95 % CI) [p-value)	Malaria^2^ β (95 % CI) [p-value)	Geohelminths^3^ β (95 % CI) [p-value)	Bacterial Pneumonia^4^ β (95 % CI) [p-value)	Kaposis sarcoma^5^ β (95 % CI) [p-value)
Time*	−0.017	−0.018	−0.003	−0.01	0.004
	(−0.0172 to −0.0168)	(−0.018 to −0.017)	(−0.003 to −0.002)	(−0.01 to −0.01)	(0.0006 to to.007
	[<0.001]	[<0.001]	[<0.001]	[<0.001]	[0.020]
**Sex**					
**Female**	1	1	1	1	1
**Male**	0 .0026	−0.061	−0.002	0.004	−0.08
	(−0.018 to 0.013) [0.743]	(−0.11 to −0.015) [,0.009]	(−0.019 to 0.014) [0.763]	(−0.039 to0.049) [0.845]	(−0.33 to 0.18) [0.544]
**Age**					
**<30 yrs**	1	1	1	1	1
**30-39**	−0.0019	0.09	0.015	0.03	−0.26
	(−0.017 to 0.014)	(0.05 to 0.13)	(−0.001 to 0.03)	(−0.012 to 0.072)	(−0.57 to .049)
	[0.809]	[0.001]	[0.071]	[0.168]	[0.100]
**40+**	−0.0047	0.096	0.011	−0.007	−0.30
	(−0.0134to 0.023)	(0.04to 0.15)	(−0.008 to 0.029)	(−0.06 to 0.045)	(−0.70 to 0.11)
	[0.612]	[<0.001]	[0.269]	[0.789]	[0.148]
**HIV clinic**					
**Tororo**	1	1	1	1	1
**Mulago**	−0.072	0.056	0.016	0.026	−0.014
	(−0.09 to −0.05)	(0.02to 0.13)	(−0.002 to 0.035)	(−0.024to 0.077)	(−0.29 to 0.26)
	[<0.001]	[0.129]	[0.083]	[0.306]	[0.920]
**Mbarara**	−0.033	0.43	0.022	0.018	0.12
	(−0.08 to −0.017)	(0.37 to 0.49)	(0.006 to 0.039)	(−0.037to 0.073)	(−0.17 to 0.42)
	[0.001]	[,0.001]	[0.009]	[0.518]	[0.404]
**Constant (**β_**0**_ **)**	4.7	3.8	4.3	3.4	0.64
	(4.6 to 4.8)	(3.7to 3.9)	(4.2 to 4.3)	(3.3 to 3.5)	(0.27 to 1.03)
	[<0.001]	[<0.001]	[<0.001]	[<0.001]	(0.001)
**Month**					
δ^**2**^_μ_	0.0063	0.007	0.0016	0.017	0.044
**(95 % CI)**	(0.0028 to 0.014)	(0.003 to 0.019	(0007to 0.004)	(0.007 to 0.040)	(0.008 to 0.22)
**[SE]**	[0.003]	[0.003]	[0.0007]	[0.007]	[0.036]
δ^**2**^_ε_	0.071	0.26	0.087	0.179	0.32
**(95 % CI)**	(0.069 to 0.074)	(0.25 to 0.28)	(0.084 to 0.09)	(0.17 to 0.19)	(0.23 to 0.42)
**[SE]**	[0.001]	[0.007]	[0.0014]	[0.006]	[0.048]

*Time =144 months, β = beta coefficient, SE = standard error, CI = confidence interval, δ^2^_ε_ = Residual variance

δ^2^_μ_ = random effect monthly variance

^1^Natural log monthly prevalence (per 1000) for Diarrhoea (<1 month)

^2^Natural log monthly prevalence (per 1000) for Malaria

^3^Natural log monthly prevalence (per 1000) for Geohelminths

^4^Natural log monthly prevalence (per 1000) for Bacterial Pneumonia

^5^Natural log monthly prevalence (per 1000) for Kaposis sarcoma

Diarrhoea (<1 month) monthly prevalence trend exhibited a curvilinear decrease with time, declined rapidly in the first 12 months (45 %) and thereafter levelled off in the following months (Fig. 1). However, after log transformation, monthly prevalence decreased linearly with time (R^2^ = 0.79, β = −0.02, p < 0.001) and negative correlation coefficient of 0.89 was found in Pearson’s correlation between calendar time and prevalence of diarrhoea (Fig. 2). Diarrhoea mean annual prevalence reduced by 83 % (12 % to 2 %) between 2002 and 2013 and the largest decline was in 2003 (45 %). The mean annual prevalence showed a signicant reducing trend (X^2^_trend_ = 177.6, P < 0.001) (Table 4). After adjusting for age, sex and clinic as fixed effects and monthly clustering as a random effect in a mixed effects linear regression model, average monthly prevalence declined at a rate of 2 % per month (p < 0.05). The rate of decline per month did not differ significantly by age and sex (p > 0.05) but differed significantly by clinic (p < 0.05) (Table 5). The monthly rate for Mulago HIV clinic in central Uganda declined 9 % times more compared to that of Tororo HIV clinic in Eastern Uganda(p < 0.001) whereas the monthly rate for Mbarara HIV clinic in western Uganda declined 0.03 times that of Tororo HIV clinic after adjusting for age and sex.

Bacterial pneumonia had an increasing monthly prevalence trend in the first 24 months (2002–2003) and thereafter monthly prevalence declined gradually (Fig. 1). A negative correlation coefficient of 0.52 was found in Pearson’s correlation between calendar time and prevalence of bacterial pneumonia (Fig. 2). Bacterial pneumonia mean annual prevalence reduced by 56 % (3.2 % to 0.7 %) between 2002 and 2013 and the largest impact was in 2010 (41 %) The mean annual prevalence for bacterial pneumonia showed a signicant reducing trend (X^2^_trend_ = 21.7, P < 0.001) (Table 4). The peak prevalence was in 2003 at (32/1000) but the largest reduction was between 2009 and 2010(41 %) (Table 4). Generally monthly prevalence did not decrease consistently but was characterised by increasing and decreasing trends over time. Hence the net monthly decrease was small (R^2^ = 0.27, β = −0.01, p < 0.001) (Fig. 2). After adjusting for age, sex and clinic as fixed effects and monthly clustering as a random effect in a mixed effects linear regression model, average monthly prevalence of bacterial pneumonia declined at a rate of 1 % per month (p < 0.05). The rate of change per month did not differ significantly by age, sex and clinic (p > 0.05) (Table 5).

The sum of the mean number of reported malaria cases per year was 3471 of which 309 (8.9 %) were confirmed malaria cases and 3162 (91.1 %) were suspected cases treated for malaria. However only confirmed cases were considered for analysis. Malaria monthly prevalence trends exhibited a curvilinear decrease with time, decreased rapidly in the first 12 months from 8 % to 2 % (80 %) and thereafter levelled off in the following months (Fig. 1). A negative correlation coefficient of 0.75 was found in Pearson’s correlation between calendar time and prevalence of malaria (Fig. 2). Malaria mean annual prevalence reduced by 87 % (8 % to 1 %) between 2002 and 2013 and the largest impact was in 2003 (80 %). The mean annual prevalence showed a signicant reducing trend (X^2^_trend_ = 102.6, P < 0.001) (Table 4). After adjusting for age, sex and clinic as fixed effects and monthly clustering as a random effect in a mixed effects linear regression model, average monthly prevalence declined at a rate of 2 % per month (p < 0.05) (Table 5). The monthly rate differed significantly by age, sex and clinic (p < 0.05). Malaria prevalence declined by 6 % times more in men compared to women (p = 0.01) after adjusting for age and clinic. The monthly rate also declined 9 % more in those aged 30-39years and 10 % more in those aged 40 years and above as compared to those <30 years (<0.001) after adjusting for sex and clinic. However the monthly rate of decline for Mulago HIV clinic was not significantly different from that of Tororo HIV clinic (p > 0.05) but the monthly rate for Mbarara HIV clinic was significantly different from that of Tororo HIV clinic (p < 0.05) after adjusting for age and sex) (Table 5).

Geohelminths included *Ascaris lumbricodes*, *Trichuris trichiura*, *Necator americanus*, *Strongyloides stercoralis* and *Ancylostoma duodenale*. Overall the monthly prevalence was characterised by increasing and decreasing trends, first increased between 0–12 months, then declined between 12-60months, then increased again between 60-96months and thereafter declined for the rest of the remaining study period (Fig. 1). A negative correlation coefficient of 0.32 was found in Pearson’s correlation between calendar time and prevalence of geohelminths (Fig. 2). Geohelminthic infection mean annual prevalence reduced by 56 % (9 % to 4 %) between 2002 and 2013 and the largest impact was in 2005 (34 %) The mean annual prevalence showed a signicant reducing trend (X^2^_trend_ = 6.2, P = 0.01) (Table 4). The peak prevalence was in 2003 at (88/1000) but the largest reduction was between 2004 and 2005(34 %) (Table 4). Because monthly prevalence did not decrease consistently but was characterised by increasing and decreasing trends over time, the net monthly decrease was small (R^2^ = 0.10, β = −0.03, p < 0.001) (Fig. 3). After adjusting for age, sex and clinic as fixed effects and monthly clustering as a random effect in a mixed effects linear regression model, average monthly prevalence declined at a rate of 0.3 % per month (p < 0.05) (Table 5). However, the rate of change per month did not differ significantly by age, sex and clinic (p > 0.05)

Kaposis sarcoma was found very rare (Fig. 1) and a showed non-significant mean annual increasing trend (X^2^_trend_ = 1.67, P = 0.20) (Table 4). A positive correlation coefficient of 0.26 was found in Pearson’s correlation between calendar time and prevalence of Kaposis sarcoma (Fig. 2). After adjusting for age, sex and clinic as fixed effects and monthly clustering as a random effect in a mixed effects linear regression model, average monthly prevalence increased at a rate of 0.4 % per month (p < 0.05). However, the rate of change per month did not differ significantly by age, sex and clinic (p > 0.05) (Table 5)

## Discussion

Diarrhoea has for long been reported to be one of the commonest complication in HIV positive individuals associated with opportunistic enteric infections [23]. Previous studies show up to 60 % of people living with HIV experience diarrhoea, that negatively affects their quality of life and adherence to HAART [24]. Diarrhoea among HIV positive individuals may be due to multiple causes including infectious causes (bacterial, viral, protozoal, heliminthic, etc.) or non-infectious causes(ARV drug effects e.g. ritonavir-boosted protease inhibitors such as Lopinavir/ritonavir or nelfinavir) [24–28]. Its prevalence is significantly higher among HIV-positive people when compared to matched controls [24]

A previous study in Uganda reported the commonest causes of diarrhoea to be helminthic infections(29.5 %), bacterial infections (19.2 %) and protozoal infections (9.2 %) [29]. Enteric viruses have also been reported associated with diarrhoea [23]. In the current study, diarrhoea prevalence declined from 12 % in the period before HAART to 2 % in 2013. In fact the biggest decline in diarrhea prevalence happened in 2003(45 %) before HAART was introduced. This was proabably due to the introduction of cotrimoxazole prophyalaxis in 2003. Our findings are consistent with other previous studies that examined the effect of cotrimoxazole prophylaxis on diarrhoea. A study in Eastern Uganda found a significant reduction in the incidence of diarrhoea after 18 months of cotrimoxazole prophylaxis(RR = 0.65; 95%CI = 0.53-0.81; p < 0.0001) [6]. Trends of bacterial diarrhoea in persons infected with HIV in the USA from 1992 to 2002 also showed a significant decrease in the period after HAART compared to the period before HAART (OR 0.4, 95%CI 0.2-0.6) [30]. The persitence of diarrhoea in spite of the increassing access to HAART and universal cotrimoxazole prophylaxis is most likely due to non-infectious causes as has previously been reported [24, 31]. A cohort study in Uganda that followed children and adolescents aged 6 weeks to 18 years who were ART for 5 years(2004–9) reported 13.% had diarrhoea attributed to ART use [31]. With global scale up of HAART access, the incidence of infectious diarrhoea has decreased but incidence of non-infectious diarrhoea has increased. More studies are urgently required to give more insight on how we can overcome this challenge and improve the quality of life for persons living with HIV/AIDS.

In Uganda, malaria is still the leading cause of illness and death particularly among the vulnerable groups including pregnant women, children under five years and people living with HIV/AIDS. In recent years, tremendous progress has been made in the fight against malaria through various interventions including increased public awareness about preventive measures; increased access to insecticide treated nets, introduction of rapid diagnostic tests (RDTs) and improved anti-malarial treatment. In this study we have observed a steep decline in malaria prevalence in 2003(80 %) which levelled off in the following years. This decline could partly be attributed to the introduction of universal cotrimoxazole prophylaxis for all HIV positive patients in Uganda in 2003 [32]. This finding is consistent with previous studies that reported a lower risk of malaria after cotrimoxazole prophylaxis compared to before (p = 0.02) [33]. A study in Eastern Uganda, showed 72 % decrease in the rate of malaria among HIV positive individuals attributed to cotrimoxazole prophylaxis[6]. Another study in Uganda showed that ART associated with daily cotrimoxazole was more protective against malaria compared to daily cotrimoxazole alone, when administered to HIV-infected adults [34]. Many studies have also reported antimalarial treatment effect of antiretroviral drugs [35–37]. This could perhaps explain the observed low malaria prevalence after the introduction HAART. However, our estimated prevalence could be an underestimate because of excluding clinically diagnosed cases that did not get a laboratory confirmation. Additionally, the decline in malaria prevalence over time could also have been caused by the other malaria interventions in Uganda including massive distribution of insecticide treated mosquito bed nets and introduction of indoor residual spraying especially in Northern Uganda [38].

Soil transmitted helminths are endemic in sub-Saharan Africa but very little has been written about their association of with HIV infection and let alone their trends. A few previous studies showed a significant association between HIV and geohelminthic infections though this association was not consistent for all helminths. A study in Honduras found a strong association between *Strongyloides stercoralis* and HIV infection but lower risk for infections of *Giardia lamblia*, *Ascaris lubricoides* and *Trichuris trichiurias* [39]. Another study found a high prevalence of *Giardiasis* and *Strongyloidiasis* among HIV-positive patients in Brazil [40]. A similar study in Ethiopia also found higher prevalence of *Strongyloides stercoralis* among HIV-positive patients with CD4 count <200 cells/μl [41]. All these point to the fact that a reduced immunity increases the risk of infection by helminthic infections and anything that improves this immunity would ideally reduce the risk of infection. In the current study prevalence of helminthic infections remained relatively higher compared to other opportunistic infections and was less affected by the expanded coverage of HAART with time. More studies are required to have more insight on the effect of HAART on severity of infection and risk of new geohelminthic infections.

Bacterial pneumonia is one of the commonest respiratory infections associated with HIV/AIDS in Africa [42]. Whereas pneumocystis pneumonia (PCP) is common in European countries and the United states but is uncommon in Africa [43]. Community-acquired bacterial pneumonia caused by *Streptococcus pneumoniae* is one of the commonest respiratory tract infections in persons living with HIV/AIDS [44]. Though preventable, bacterial pneumonia is one of the leading causes of morbidity and mortality among persons living with HIV/AIDS in many sub-Saharan Africa countries [45–48]. A review of studies on HIV/AIDS related opportunistic infections in sub-Saharan Africa show high prevalence of *S. pneumonia* infection ranging from 25 % in Cameroon to 31 % in Uganda [47]. In the current study, prevalence of bacterial pneumonia showed an increasing trend up to 2003, and thereafter reduced consistently with increasing access to HAART. This is probably due to universal cotrimoxazole prophylaxis introduced in 2003 and HAART in 2004. Some studies have shown HIV infection to be associated with a 10-fold increase in incidence of bacterial pneumonia [42]. However our findings are consistent with other previous studies which showed a significant reducing effect of cotrimoxazole prophylaxis and HAART. For instance a study Kohli et al. showed HAART and cotrimoxazole prophylaxis were associated with an 8 % monthly risk reduction of bacterial pneumonia [49]. However our findings differs from those obtained in a study in the USA that evaluated annual trends for 13 most common AIDS-defining opportunistic infections by examining medical records in more than 90 hospitals and clinics in 9 US cities before HAART (1991–96) that showed decreasing trend in all the OIs except pneumonia [50]. Another study in the USA reported high rates of bacterial pneumonia among HIV-positive women compared to HIV-negative women [49]. The rate of bacterial pneumonia among HIV-infected women was 8.5 cases per 100 person-years, compared with 0.7 cases per 100 person-years in HIV-uninfected women (*P* < .001). Relatively few studies have characterised the impact of HAART on bacterial pneumonia in resource poor settings and more studies are therefore recommended.

Kaposis’s sarcoma which is an AIDS-related malgnancy had no evidence of a significant trend. Kaposi’s sarcoma usually associated with advanced HIV disease was found rare in our study. Though strictly speaking, it’s not an opportunistic infection but was studied because of its close association with viral infections (human herpes virus type 8) [12]. In developed countries, the introduction of highly active antiretroviral therapy (HAART) in 1996 led to a tremendous decrease in the incidence of Kaposi’s sarcoma (KS) among people with HIV or AIDS (PWHA) [51]. Our findings are consistent with another study in Kenya in which medical records at Nairobi hospital were reviewed for Kaposis sarcoma covering the period 1968 to 1997 and was found to be very rare at only 2-5 % [52]. A retrospective review of clinical records in a teaching hospital in North-western Nigeria covering the period 1994–2004 also found the prevalence of Kaposis sarcoma to be very low (only 27cases in 11 years) [53]. Another study that examined trends of Kaposis sarcoma for the period 1982–2009 in Brazil found a prevalence of only 2.4 % among HIV+ individuals [54]. The study also showed a significant reduction in Kaposis sarcoma prevalence attributed to increased access to HAART over time [54]. Another study that evaluated time trends of AIDS-defining diseases diagnosed in Italy during the pre-HAART period (1982–1996) noted downward trends for mycoses, PCP, Karposis sarcoma and non-Hodgkin’s lymphomas and upward trends for mycobacterioses, bacterial and protozoal infections [55]. However, more studies giving insight on the impact of HAART on Kaposis sarcoma and other HIV-related opportunistic cancers in resource poor settings are still required.

### Limitations

This study had a number of limitations. First, data analysed was limited to what was available in the TASO electronic data base and so certain variables whose data was not captured could not be analysed. Being a retrospective cohort, there was no information available on the clients who never returned for care. So we did not have information on the connection to care or survival status of clients who were lost to follow up. However we assumed that these simply relocated to another clinic for care or died as had been reported in other previous studies [56–60]. Even from sensitivity analysis (results not shown), we established that cases who remained in care were not significantly different from those that were lost to follow up and so we conservatively assumed that cases who remained in care were a representative sample of all the cases in the original cohort and that missing clinic visits happened randomly. The TASO clients whose medical records were analysed may not represent all HIV positive individuals in Uganda which means generalisability could be limited but still this does not compromise the evidence we have adduced since most if not all beneficiaries of public health care programmes in Uganda are generally the same. There may also have been a possibility that some OIs went away undiagnosed or were not recorded during clinic visits as was reported in a previous study conducted in Uganda [61]. Though we believe this was minimized by selected OIs which were easy to diagnose and could have been a small number which may not have significantly influenced the overall trends. The decreasing trends of OIs could partially be related to immune recovery due to HAART. However CD4 monitoring data for most patients were not available probably because of being unaffordable in these resource poor settings. Additionally, trends in the reported disease conditions needs to be compared with trends in HIV-negative controls since these conditions are not exclusively limited to HIV-infected individuals. This would have helped to explain better whether the decline in trends could only be attributed to HAART and/or co-trimoxazole prophylaxis. Even with these limitations, there is sufficient evidence that increasing HAART availability in resource poor settings is having a meaningful impact on prevalence of opportunistic infections.

## Conclusions and recommendations

Our findings show a declining trend in the prevalence of most OIs, though some like geohelminths remain an important public health problem even in the era of HAART. The differences in trends between clinics were likely to be due to differences to exposure to infectious agents and the rural/urban disparity. Tororo being most remote and rural had the highest prevalence of the OIs studied and needs special consideration for targeted interventions. With an increasing number of HAART eligible HIV-infected patients amidst limited resources to sustain lifelong access to treatment, more studies are needed to assess the effectiveness of current treatment strategies on the quality of life of persons living with HIV/AIDS to better inform policy and planning of HIV/AIDS health care service. Future studies should compare trends in HIV positive individuals with trends in HIV negative controls to better understand whether the declining trends are exclusively due to increasing access to HAART and co-trimoxazole prophylaxis. There is also urgent need to integrate interventions targeting malaria and geohelminths in HIV programmes.
